# Calomyscid Rodents (*Rodentia*: Calomyscidae) as a Potential Reservoir of Zoonotic Cutaneous Leishmaniasis in a Mountainous Residential Area in the Plateau of Iran: Inferring from Molecular Data of kDNA and ITS2 Genes of *Leishmania Major*

**DOI:** 10.1155/2023/5965340

**Published:** 2023-02-10

**Authors:** Saeed Shahabi, Kourosh Azizi, Qasem Asgari, Bahador Sarkari

**Affiliations:** ^1^Department of Parasitology and Mycology, School of Medicine, Shiraz University of Medical Sciences, Shiraz, Iran; ^2^Department of Biology and Control of Disease Vectors, School of Health, Shiraz University of Medical Sciences, Shiraz, Iran; ^3^Basic Sciences in Infectious Diseases Research Center, Shiraz University of Medical Sciences, Shiraz, Iran

## Abstract

Cutaneous leishmaniasis (CL), a neglected tropical disease, is an important health problem in Fars Province, southern Iran. Fars, the fourth most populous Province in Iran, is the center of both anthroponotic and zoonotic cutaneous leishmaniasis (ZCL). Rodents, the reservoir of *Leishmania major*, play an important role in transmitting ZCL. In the present study, we report *Leishmania* infection in calomyscid rodents for the first time in mountainous residential areas of Shiraz, the capital of Fars Province, in southern Iran. Rodents were trapped in urban mountainous areas. The skin, liver, and spleen of rodents were examined microscopically for *Leishmania* infection. In addition, DNA was extracted from the tissues and they were evaluated for *Leishmania* infection by targeting the kDNA and subsequent sequencing of the nuclear rDNA internal transcribed spacer two (ITS2) region. DNA of *L. major* was detected in the spleen and liver of calomyscid rodents. Molecular evolution based on DNA-sequencing of the ITS2 gene confirmed the taxonomic situation of the parasite as *L. major*. Our findings suggest the eco-epidemiological importance of calomyscid rodents in the foci of leishmaniasis in the mountainous residential area on the plateau of Iran. These rodents may play a role in the transmission of leishmaniasis in a residential area and could be considered a potential reservoir for CL.

## 1. Introduction

Cutaneous leishmaniasis (CL) is caused by infection with protozoan parasites belonging to the genus *Leishmania* (Kinetoplastida: Trypanosomatidae). Infected female sandflies belonging to the subfamily phlebotomine act as the main vectors of these parasites and transmits these parasites to humans through biting. CL present as skin lesions, mainly ulcers, on exposed parts of the body, leaving life-long scars and serious disability or stigma. About 95% of CL cases occur in the Americas, the Mediterranean Basin, the Middle East, and Central Asia [1]. According to the WHO annual report, leishmaniasis is one of the most neglected tropical diseases, which remains a major public health problem worldwide and is classified as category I by the TDR/WHO, mainly due to the absence of applicable control measurements [2].

Iran is one of the endemic countries wherein many new CL cases occur each year [3–6]. Fars Province, located in the south of Iran, is one of the main foci of visceral and cutaneous leishmaniasis. Based on a recent study about the geographical distribution and molecular epidemiology of CL in Fars Province [7], both anthroponotic cutaneous leishmaniasis (ACL) and zoonotic cutaneous leishmaniasis (ZCL) were detected in Shiraz city, while in most other counties of the Province only ZCL has been reported based on molecular data [7]. Therefore, Shiraz city, which has a population of over 1.5 million people, is of special importance as one of the foci of leishmaniasis.

*Rodentia* is the most diverse mammalian order in Iran. These animals are considered reservoirs of various zoonotic diseases including parasitic infections [8]. Various species of rodents in Iran and Fars Province have been identified as the reservoir or potential reservoir hosts of *L. major*, the agent of ZCL [9–12]. The rodents of Fars Province belong to seven families including the Calomyscidae family [13].

The family Calomyscidae with the only genus of *Calomyscus* was considered a monospecies with the only species *C. bailwardi* for a long time [14–17]. In the recent revisions, more than eight species have been considered for the genus [13, 18–25]. The members of this family were formerly classified as long-tailed or mouse-like hamsters in the Cricetinae subfamily of Cricetidae and then elevated to the subfamily of *Muridae* [26]. However, these rodents are not related to true hamsters and represent an ancient branching of the muroid superfamily [26]. In the last revision of the taxonomy of these rodents [27], the genus *Calomyscus* was placed in a separate family of Calomyscidae in the superfamily of *Muroidae*.

These nocturnal rodents inhabit barren, rocky hills in the dry parts of their range, and hillsides covered with trees of evergreen oaks, pistachio (*Pistacia atlantica*), and mountain almonds (*Amygdalus scoparia*) in Syria, Azerbaijan, Iran, Turkmenistan, Afghanistan, Pakistan, and Turkey [23, 28]. Most *Calomyscus* species inhabit the Zagros and Elburz Mountains of Iran including the mountainous area of Fars Province and Shiraz city [13, 23]. Recent studies conducted in different parts of the Fars Province show that the predominant species of the parasite causing the CL is *L. major*, and the disease has spread in recent years from rural to suburban and even within the city, which could lead to an unexpected outbreak of the disease. The study of different epidemiological features of the foci is very important to timely identify vectors, reservoirs, and the type and genetic characteristics of the parasite in order to take the necessary measures for controlling the disease in this urban area. Accordingly, the present study was conducted to investigate the possible infection with *Leishmania* infection in rodents in the current emerging foci of CL in Shiraz, the capital of Fars Province, southern Iran.

## 2. Materials and Methods

### 2.1. Area of Study

**Figure 1:**
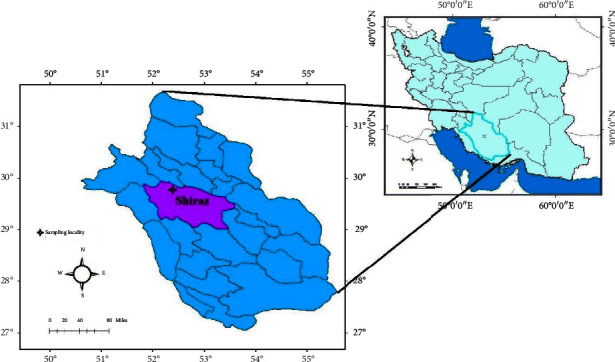
Map of Fars Province of Iran shows rodents' sampling locality (star symbol) in the newly emerging focus of leishmaniasis in the city of Shiraz. The sampling areas were not too far from each other and were shown as one location on the map.

The study was conducted in Shiraz city, the capital of Fars Province (Figure 1), which is the fourth-largest Province of the country with a population of nearly 5 million people, located in the south of Iran. Shiraz is an important historical, cultural, social, and economic city in southern Iran [29]. It is the third-largest city and the fifth-most-populous city in Iran, which is also known as Pars and Persis (Persia). The city with an average elevation of 1500 m above sea level is located in an NW–SE elongated valley bounded by the Zagros Mountains. Shiraz has a moderate climate with four regular seasons. The daily temperature varies approximately between 40°C in the summer and 10°C in the winter [29].

### 2.2. Rodent Capturing and Tissue Sampling

Rodents were trapped in the north of Shiraz (Figure 1) in the mountainous area near buildings in which leishmaniasis patients were living, using Sherman live traps baited with a piece of cucumber, walnut, and cheese puffs. The traps were set in the evening and collected the next morning. Rodents were transferred to the animal laboratory of Shiraz University of Medical Sciences for further examination. Rodents were euthanized with a fixed flow rate of CO_2_ (20% chamber replacement rate [CRR]). Then rodents were photographed, and sex, characteristics, and external measurements (lengths of body, ear, hindfoot, and tail) were recorded and carefully examined for skin lesions. After that, the animals were dissected, and impression smear samples were taken from the liver, spleen, ears, and sole. Smears were stained with Giemsa's stain and examined microscopically for the presence of *Leishmania* amastigotes. Moreover, samples of the spleen, liver, and lesion of the skin were taken in pure ethanol for subsequent DNA extraction. After removing the viscera, the remaining body was kept in alcohol for further morphological identification.

### 2.3. DNA Extraction and Polymerase Chain Reaction (PCR)

Genomic DNA was extracted from the blood and tissue samples including the liver, spleen, ears, and sole, using a DNA extraction Kit (Favorgen Biotech, Taiwan) according to the manufacturer's instructions. The final DNA was eluted in a 50 *μ*L of elution buffer and kept at −20°C until use [30].

For amplification of a specific fragment of the kDNA gene in *Leishmania*, the two primers of LIN4R (F): 5′- GGG GTT GGT GTA AAA TAG GG -3′ and LIN17 (R): 5′- TTT GAA CGG GAT TTC TG -3′ were used. The primers amplified 650-bp and 760-bp kDNA fragments of *L. major* and *L. tropica*, respectively [7].

Also, the *Leishmania* ITS2 gene was amplified, using pair primers of 5′-AAACTCCTC TCTGGTGCTTGC-3′ (forward) and 5′-AAACAAAGGTTGTCGGGGG-3′ (reverse). The thermal cycling conditions were as follows: denaturing at 94.5°C for 5 min, 35 cycles of denaturing at 94°C for 30 s, annealing at 55°C for 30 s, extension at 72°C for 30 s, followed by a final extension at 72°C for 8 min. PCR amplification of both genes, was carried out in a final volume of 25 *μ*L, containing 0.6 *μ*L (10 pm) of each primer, 1 *μ*L of extracted DNA (100 ng/*μ*L), 12.5 *μ*L of 1x Taq DNA Polymerase Master Mix RED, and 10.3 *μ*L of distilled water (DW).

The PCR products (3.5 *μ*L) along with 3.5 *μ*L of 100-bp molecular marker (SMOBIO, Hsinchu, Taiwan) were subjected to electrophoresis at 80 voltages for 45 min. The PCR product was observed by UV transillumination and photographed (Uvitec, Cambridge, UK). Reference strains of *L. tropica* (MHOM/IR/89/ARD-L2) and *L. major* (MHOM/IR/54/LV39), and DW were used as positive and negative controls, respectively.

### 2.4. Phylogenetic Analyses

The raw nucleotide sequence with both forward and reverse directions were visually checked and analysed, using the Chromas program as implemented in the software BioEdit version 7.2.5 [31], and the consensus sequence was created. The final sequence was submitted to GenBank (NCBI) with the accession number ON398770. The consensus sequence was compared to reference sequences in NCBI and identified by BLAST similarity search, using the BLAST algorithm. The present sequence and other reference sequences derived from NCBI were aligned, using Clustal W, as implemented in the software BioEdit version 7.2.5 [31], and then converted in FASTA format for phylogenetic analyses in MEGAX software [32]. The analyses involved 14 ITS2 gene sequences including 13 sequences of *Leishmania* and one sequence of *Crithidia mellificae* as an outgroup. A total of 12 ITS2 gene sequences as references of *Leishmania* species were selected from the GenBank database as follows: one sequence of *L*. *infantum* (MW534746), one *L. donovani* (FJ753386), one *L. tropica* (FJ948464), five *L. major* (AJ786165, MT497959, MT497977, DQ300195, and MN931859), one *L. chagasi* (GU045591), one *L. amazonensis* (MT940882), one *L. mexicana* (FJ948432), and one *L. aethiopica* (GQ920673). There were a total of 494 positions in the final dataset. Phylogenetic relationships between *Leishmania* species were reconstructed using the maximum likelihood (Tamura-Nei model [33]), neighbor-joining [34], and UPGMA methods [35] in MEGA X [32]. In the maximum likelihood method, algorithms of neighbor-join and BioNJ to a matrix of pairwise distances estimated using the maximum composite likelihood (MCL) approach were applied to obtain initial trees for the heuristic search. In the neighbor-joining and UPGMA methods, the evolutionary distances were computed using the Jukes–Cantor method [36]. Bootstrap test (10000 replicates) based on the percentage of replicate trees in which the associated taxa clustered together was shown next to the branches.

## 3. Results

**Figure 2:**
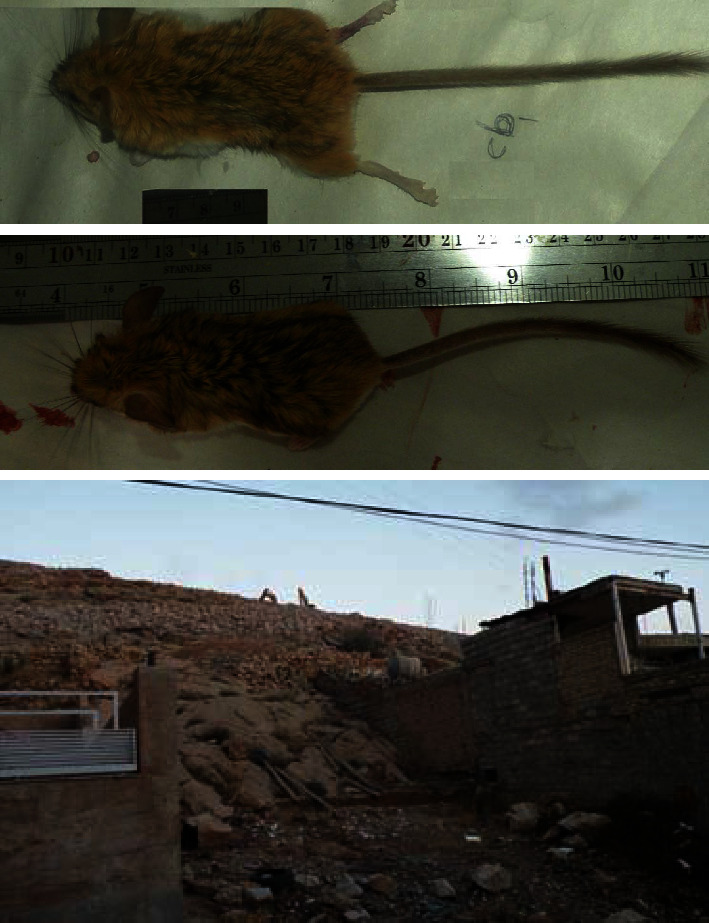
Up: *Calomyscus cf. bailwardi* infected by *L. major* captured from Shiraz; down: rodent habitats destroyed due to road construction.

**Figure 3:**
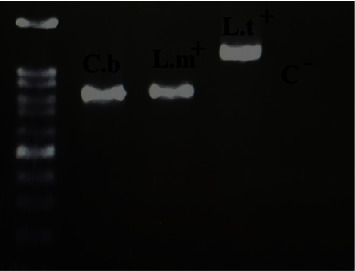
The results of the PCR-based amplification of kinetoplast DNA recovered from the spleen tissue sample from *Calomyscus cf. bailwardi* (C. b). Reference samples of *L*. *tropica* (L. t+) and *L. major* (L. m+) and negative control (C−) were run in other lanes.

**Figure 4:**
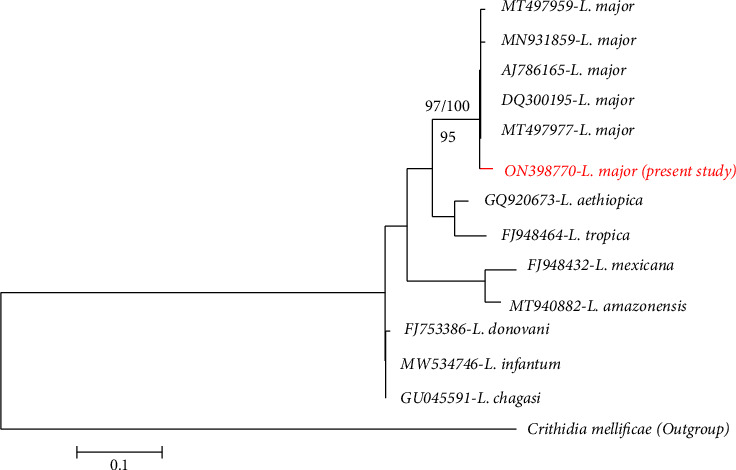
Evolutionary relationships of *Leishmania* species based on the sequences of ITS2 gene showing the *L. major* infecting *Calomyscus cf. bailwardi* in the present study (accession number of ON398770), inferred using the neighbor-joining, UPGMA, and maximum likelihood methods. The percentage value of the bootstrap test is shown next to the branches. Nodal supports presented above the line indicate neighbor-joining (left) and UPGMA bootstrap (right) with 100000 replicates using MEGA X The numbers below the line represent bootstrap support values for maximum likelihood (500 replicates).

All 10 Calomyscid samples were identified as *Calomyscus cf. bailwardi* (Figure 2). While *Leishmania* amastigotes were not seen in the smear samples taken from the spleen, liver, blood, ears, and sole, *Leishmania* DNA was detected in the blood, liver, and spleen samples of one out of 10 trapped *Calomyscus,* based on PCR of the kDNA gene (Figure 3) and also DNA-sequencing of the ITS2 gene (Figure 4).

BLAST analysis revealed that the parasite infecting *Calomyscus cf. bailwardi* had 97.5 to 100% (45 to 100% coverage) identity to *L. major* sequences of the ITS2 region. All phylogenetic reconstruction based on UPGMA, maximum likelihood, and neighbor-joining methods also revealed the parasite infecting *Calomyscus cf. bailwardi* belonged to *L. major* (Figure 4) supported with bootstraps of 95 to 100 shown at the base of the clade. The clade included *L. major* sequences downloaded from NCBI.

## 4. Discussion

In Iran, especially in Fars Province, gerbil rodents (subfamily Gerbillinae) have been frequently reported and considered as the main reservoirs of ZCL [10–12, 37]. Furthermore, other murid species like *Mus musculus* [38], *Rattus norvegicus* [39], *Acomys dimidiatus* [9], and *Nesokia indica* [40] have been identified as potential reservoir hosts of the *L. major* in the country. Nevertheless, until now, there has not been any information about the infection of calomyscid rodents (Family: Calomyscidae) with *Leishmania* species.

However, the status of rodents as reservoirs in the urban mountainous area of Shiraz, a populated city in Iran, remains unclear. The new focus of leishmaniasis in the present study encompasses partly a residual mountainous area where the calomyscid rodent inhabits. Here, we demonstrated natural *Leishmania* infections in calomyscid tissues, showing that these rodents may play a role in the transmission of leishmaniasis in this mountainous area. Another factor in the outbreak of the disease in this area is rodents' habitat destruction due to road construction in the area, which has caused the influx and movement of these rodents and other rodents toward residential areas. Since there is a big area of gardens, other rodents like *Rattus rattus* and *Mus musculus* can also be infected by *L. major* [38, 41–43]. Therefore, leishmaniasis in this area is linked to environmental change and urbanization in the mountainous area. Calomyscid rodent infection may have occurred through the transmission of *L. major* either from other rodents or from infected humans through Phlebotomus sand flies. The natural infection of these rodents to *L. major* should be examined by further studies on other foci in the mountainous area in Iran. Also, the study of *Leishmania* infection in sandfly mosquitoes is one of the prerequisites of any CL control program in such areas.

## 5. Conclusion

Our findings suggest the eco-epidemiological importance of calomyscid rodents in the foci of leishmaniasis in the mountainous residential area on the plateau of Iran. These rodents may play a role in the transmission of leishmaniasis in a residential area and could be considered a potential reservoir for CL.

## Data Availability

Data used to support the findings of this study are included in the article.
